# The association between young age at metastatic breast cancer diagnosis and overall survival in the EMBRACE study

**DOI:** 10.1038/s41523-025-00822-y

**Published:** 2025-08-29

**Authors:** Kristen D. Brantley, Gregory J. Kirkner, Melissa E. Hughes, Leticia Varella, Georgia Suggs, Olivia M. Cunningham, Sanjana Ravikumar, Craig Snow, Sara M. Tolaney, Sarah Sammons, Ann H. Partridge, Nancy U. Lin

**Affiliations:** 1 Department of Medical Oncology, Dana-Farber Cancer Institute, Boston, MA USA; 2 Department of Medicine, Harvard School of Medicine, Boston, MA USA; 3 Breast Oncology Program, Dana-Farber Brigham Cancer Center, Boston, MA USA; 4 Yale Cancer Center, New Haven, CT USA

**Keywords:** Breast cancer, Breast cancer

## Abstract

The influence of young age at diagnosis on prognosis of patients with metastatic breast cancer (MBC) remains unclear. We examined overall survival (OS) within a single-institution prospective study of patients with de novo or recurrent MBC. Kaplan-Meier curves assessed OS by age (≤35 or ≤40 years as the youngest category) and inferred metastatic tumor subtype. Multivariable Cox regression models estimated hazard ratios (HRs) and 95% CIs for OS by age adjusting for clinical factors. Of 4189 women <75 years, 571 were ≤40 years at MBC diagnosis, of whom 260 were ≤35 years. Over half (52%) died during follow-up (median = 5.3 years, IQR = 2.1–9.8 years). Compared to patients 45–55 years, those ≤35 years at diagnosis experienced worse OS (HR = 1.22, 95%CI 1.00–1.48, *p* = 0.05). This association was driven by HER2-negative/luminal B-like and hormone receptor-positive/HER2-positive tumors. These findings highlight the need to develop more effective therapies for young patients with this metastatic subtype.

## Introduction

The majority of breast cancer (BC)-related deaths are due to metastatic disease^1^. In the U.S., de novo metastatic BC (MBC) accounts for approximately 3–6% of incident BC cases^2^ and roughly 30% of all patients with early-stage BC will develop recurrent MBC^3^, with rates dependent on primary BC subtypes and treatments^4,5^. The high mortality rates of patients with MBC^6,7^, especially for those with recurrent MBC^8–10^, indicate the need for further exploration of predictors of overall survival (OS) in MBC.

Studies of patients with early-stage BC have demonstrated prognostic differences by age at diagnosis, with recent studies indicating worse BC-specific survival among young patients (≤40 years) compared to those older at diagnosis^11,12^, dependent on molecular subtype^12,13^. While younger women are more likely to be diagnosed with de novo MBC and have approximately double the risk for recurrent metastasis compared to their older counterparts^5,14^, the association between age at MBC diagnosis and OS is unclear. Studies report both better^15–17^, and worse OS^5,18^ for young patients with MBC, which may be attributed to heterogeneity between the represented patient cohorts, differences in the definition of young age (including <50 years,^15^ <40 years,^17^ ≤35 years^18^, and continuous age^16^), and inconsistent sets of adjustment factors used in multivariable models.

Studies of registry-based data rarely capture recurrence, leaving the largest studies on OS of MBC generalizable to the minority of patients who present with de novo MBC. Tumor molecular subtype is a known prognostic factor^19,20^ that differs in frequency and in association with OS by age at diagnosis^12,21,22^. However, most studies adjust for subtype by using hormone receptor and human epidermal growth factor receptor 2 (HER2) receptor status alone^15–17^, lacking the important distinction between luminal A and luminal B subtypes. In addition, most studies examining OS in patients with MBC have relied on primary as opposed to metastatic tumor subtype, misclassifying those individuals who will develop a metastasis with a different (often more aggressive) subtype than their primary tumor^23^, and limiting interpretation given that clinical recommendations emphasize treatment-based decision-making by metastatic tumor characteristics^24^. Here we evaluated whether and to what extent age influences survival following MBC diagnosis, accounting for clinicopathologic features of metastatic tumors.

## Results

### Patient characteristics

After excluding 31 women with unknown vital status and 178 women ≥75 years at metastatic diagnosis, the analytic cohort included 4189 patients, enrolled between 2009–2023, with diagnosis of MBC ranging from 1983–2023 and follow-up ending in May 2024. Median age at MBC diagnosis was 54 years (interquartile ranges [IQR] = 45–62 years, range = 18–75 years), with 571 ≤40 years, 1693 41–55 years, and 1925 >55- <75 years (Table 1, Table S1). Targeted germline genetic testing results were known for 2,610 participants (N = 62%), though numbers differed by gene tested. Among those with testing performed, a pathogenic variant (PV) in *BRCA2* was slightly more common in individuals aged 40-55 years compared to those >55–75 years (3% v. 2%, *p* = 0.05), and *BRCA1* PVs were numerically more frequent in the youngest age groups, though this was not statistically significant (2% in ≤40 years and 2% in ≤35 years v.1% in >55- < 75 years, p for ≤40 years vs. >55–75 years = 0.06). A high proportion of patients presented with stage 3 (25%) or stage 4 de novo (24%) disease. Compared to older patients, those ≤40 years at diagnosis were more likely to present with de novo disease ( ≤ 40 years: 36% v. >40–55 years: 23% v. >55-75 years: 21%, *p* < 0.001). The most common primary tumor histology was invasive ductal, though younger patients were significantly less likely to have lobular histology ( > 55 years: 18% v. ≤40 years: 5%, *p* = 0.001). Younger patients were more likely to receive radiation in the metastatic setting compared to older counterparts. Brain metastases were significantly more common in the younger patients (13%) compared to those ages 40–55 years (10%, *p* = 0.04) and those >55–75 years (*p* = 0.001).

**Table 1:** Patient and Tumor Characteristics of women <75 y at diagnosis in EMBRACE cohort (*N* = 4189)

Variable^a^	Total (*N* = 4189)	≤40 y (*N* = 571)	>40–55 y (*N* = 1693)	>55- <75 y (*N* = 1925)
Died	2190 (52%)	304 (53%)	914 (54%)	971 (50%)
Time from met dx to death or censorship, median (IQR)	2.5 (1.1–4.7)	2.3 (0.9–4.6)	2.5 (1.0–4.9)	2.6 (1.1–4.7)
Germline PV testing done	2610 (62%)	370 (65%)	1028 (61%)	1212 (63%)
Known PVs^b^				
*ATM*	15 (0.6%)	2 (0.5%)	7 (0.7%)	6 (0.5%)
*BRCA1*	40 (2%)	9 (2%)	18 (2%)	13 (1%)
*BRCA2*	59 (2%)	9 (2%)	30 (3%)	20 (2%)
*CHEK2*	31 (1%)	3 (0.8%)	14 (1%)	14 (1%)
*PALB2*	13 (0.5%)	2 (0.5%)	4 (0.4%)	7 (0.6%)
*TP53*	9 (0.3%)	3 (0.8%)	4 (0.4%)	2 (0.2%)
Other PV	62 (2%)	9 (2%)	25 (2%)	28 (2%)
Primary tumor characteristics				
Age at dx, median (IQR)	49 (41–56)	34 (30–36)	45 (41–49)	57 (52–63)
Stage				
0	57 (1%)	4 (1%)	28 (2%)	25 (1%)
1	589 (14%)	35 (6%)	213 (13%)	341 (18%)
2	1425 (34%)	170 (30%)	593 (35%)	662 (34%)
3	1031 (25%)	150 (26%)	438 (26%)	443 (23%)
4 (de novo)	1008 (24%)	204 (36%)	396 (23%)	408 (21%)
Missing stage	79 (2%)	8 (1%)	25 (1%)	46 (2%)
Histology				
DCIS	66 (2%)	6 (1%)	29 (2%)	31 (2%)
Invasive ductal	2992 (71%)	476 (83%)	1264 (75%)	1252 (65%)
Invasive lobular	535 (13%)	29 (5%)	164 (10%)	342 (18%)
Tubular	6 (0%)	0 (0%)	3 (0%)	3 (0%)
Mucinous	15 (0%)	2 (0%)	7 (0%)	6 (0%)
Micropapillary	6 (0%)	1 (0%)	4 (0%)	1 (0%)
Mixed (IDC and ILC)	357 (9%)	28 (5%)	147 (9%)	182 (9%)
Missing/Other	212 (5%)	29 (5%)	75 (4%)	108 (6%)
Tumor grade				
1	275 (7%)	12 (2%)	100 (6%)	163 (8%)
2	1618 (39%)	161 (28%)	646 (38%)	811 (42%)
3	1907 (46%)	367 (64%)	807 (48%)	733 (38%)
Missing	389 (9%)	31 (5%)	140 (8%)	218 (11%)
Subtype				
Luminal A-like^c^	1248 (30%)	103 (18%)	529 (31%)	616 (32%)
Luminal B-like^d^	1156 (28%)	181 (32%)	425 (25%)	550 (29%)
HR + /HER2+	471 (11%)	102 (18%)	214 (13%)	155 (8%)
HR-/HER2+	279 (7%)	63 (11%)	127 (8%)	89 (5%)
HR-/HER2-	675 (16%)	109 (19%)	290 (17%)	276 (14%)
Missing	360 (9%)	13 (2%)	108 (6%)	239 (12%)
Metastatic tumor and treatment characteristics				
Age at met dx, median (IQR)	54 (45–62)	36 (32–38)	49 (45–52)	63 (59–67)
Year of met dx, median (range)	2016 (1983–2023)	2016 (1996–2023)	2015 (1983–2023)	2016 (1998–2023)
Years from primary to metastatic dx, median (IQR)	2.7 (0.4–6.6)	1.2 (0–2.7)	2.5 (0.6–5.5)	3.9 (1.1–9.3)
Subtype^e^				
Luminal A-like^c^	1202 (29%)	104 (18%)	474 (28%)	624 (32%)
Luminal B-like^d^	1286 (31%)	172 (30%)	479 (28%)	635 (33%)
HR + /HER2+	427 (10%)	91 (16%)	191 (11%)	145 (8%)
HR−/HER2+	332 (8%)	70 (12%)	148 (9%)	114 (6%)
HR−/HER2-	867 (21%)	128 (22%)	374 (22%)	365 (19%)
Missing	75 (2%)	6 (1%)	27 (2%)	42 (2%)
Subtype different from primary subtype^f^	392 (18%)	45 (19%)	157 (17%)	190 (19%)
Lines of therapy from dx to last follow-up, median (IQR)	3 (1–6)	3 (1–6)	3 (1–6)	3 (1–5)
Types of metastatic therapy received, from dx to last follow-up^g^, median (IQR)				
ET	3068 (73%)	339 (59%)	1206 (71%)	1523 (79%)
CDK 4/6i	2114 (50%)	230 (40%)	820 (48%)	1064 (55%)
Chemotherapy	3881 (93%)	541 (95%)	1581 (93%)	1759 (91%)
Radiation	1759 (42%)	293 (51%)	747 (44%)	719 (37%)
Number of metastatic sites, from dx to last follow-up, median (IQR)	4 (3–5)	4 (3–5)	4 (3–5)	4 (3–5)
Sites of metastatic disease anytime from dx to last follow-up^h^, median (IQR)				
Bone	2318 (55%)	314 (55%)	916 (54%)	1088 (57%)
Brain	360 (9%)	72 (13%)	161 (10%)	127 (7%)
Liver	1109 (26%)	188 (33%)	487 (29%)	434 (23%)
Lung	814 (19%)	108 (19%)	349 (21%)	357 (19%)
Other^i^	2307 (55%)	310 (54%)	943 (56%)	1054 (55%)

*CDK4/6i* cyclin-dependent kinase 4 and 6 inhibitor, *DCIS* ductal carcinoma in situ, *dx* diagnosis, *ET* endocrine therapy, *HER2* human epidermal growth factor receptor 2, *HR* *+* hormone receptor positive, *HR−* hormone receptor negative, *IDC* invasive ductal carcinoma, *ILC* invasive lobular carcinoma, *IQR* interquartile range, *PR* progesterone receptor, *PV* pathogenic variant, *y* years.

^a^ Variable described by N (%) if not specified.

^b^ Includes pathogenic or likely pathogenic variants. Percent out of N with any germline testing in age group.

^c^ HR + /HER2- with primary tumor grade <3 & met tumor PR staining >=10%.

^d^ HR + /HER2- with primary tumor grade=3 OR met tumor PR staining <10%.

^e^ When metastatic tumor subtyping unavailable, primary tumor subtype used (*N* = 980).

^f^ Among those with recurrent MBC and with both primary and metastatic subtyping available (*N* = 2153).

^g^ Multiple types of therapy are possible for one patient.

^h^ Multiple sites of disease are possible for one patient.

^i^ Other sites reported include: bone marrow (1), duodenum (1), axilla lymph nodes (1), ovary (2), skin of breast (2), sternum (1), stomach (3), thyroid (1), uterus (2), choroid (2), endometrium (1), esophagus (2), scalp (1).

### Clinical outcomes and univariable associations of age and clinical factors with OS

Over a median follow up of 5.3 years (IQR = 2.1–9.8 years), 2190 (52%) of patients died. Among those who died, median time between metastatic diagnosis and death was 2.6 years (IQR = 1.3–4.6 years). In univariable models, age ≤35 years was associated with shorter OS compared to those >35-55 years (hazard ratio [HR] [95% CI] = 1.20 [1.01–1.43], *p* = 0.04) (Table 2), though age ≤40 years was not statistically significantly associated with OS. When comparing 10-year age groups, the difference in survival appeared most prominent between those ≤35 years v. those >35–45 years or >55–65 years (Table 2). OS was shorter among those with recurrent MBC compared to those with de novo MBC (HR [95% CI] = 1.51 [1.36–1.67], *p* < 0.001) and the reduction in OS time was stronger among those with shorter disease-free interval (DFI) (HR [95% CI]: 0–2 years v. de novo = 2.84 [2.50–3.24] v. ≥2 years v. de novo = 1.27 [1.14–1.42], *p* < 0.001) (Table 2). The strongest associations with OS were for metastatic tumor subtype and sites of metastases. Compared to luminal A-like tumors, HER2−, luminal B-like tumors were associated with approximately 2-times the hazard of death (HR = 1.85, *p* < 0.001), while HR−/HER2− tumors were associated with 4 times the hazard of death (HR = 4.22, *p* < 0.001). In addition, the hazard of death among those with brain or liver metastases was approximately 2-times that of individuals without these metastases.

**Table 2:** Univariable associations between clinical variables and OS among MBC patients

Variable	N/Deaths^a^	HR (95% CI)	*p*-value
Age group at MBC dx	4189/2190		
≤40 y		1.10 (0.97–1.25)	0.15
>40–55 y		1.00 (ref)	
>55 y		1.00 (0.92–1.10)	0.94
Age group at MBC dx	4189/2190		
≤35 y		1.20 (1.01–1.43)	0.04
>35–55 y		1.00 (ref)	
>55 y		1.00 (0.92–1.09)	1.00
Age group at MBC dx, 10 y			
≤35 y		1.16 (0.97–1.39)	0.11
>35–45 y		0.91 (0.81–1.03)	0.14
>45–55 y		1.00 (ref)	
>55–65 y		0.94 (0.84–1.05)	0.28
>65–75 y		1.01 (0.89–1.15)	0.90
Recurrent v. de novo	4110/2164	1.51 (1.36–1.67)	<0.001
Disease-free interval	4189/2190		
de novo		1.00 (ref)	
>0–24 months		2.84 (2.50–3.24)	<0.001
≥24 months		1.27 (1.14–1.42)	<0.001
Primary tumor subtype	3829/1990		
Luminal A-like		1.00 (ref)	
Luminal B-like		1.80 (1.61–2.02)	<0.001
HR + /HER2+		0.77 (0.65–0.90)	0.002
HR−/HER2+		0.92 (0.76–1.11)	0.40
HR−/HER2-		4.05 (3.57–4.59)	<0.001
Primary tumor stage			
In situ	4110/2164	1.00 (ref)	
Stage I		1.14 (0.75–1.73)	0.53
Stage II		1.45 (0.96–2.17)	0.07
Stage III		1.94 (1.29–2.92)	0.001
Stage IV (de novo)		0.99 (0.66–1.50)	0.98
Primary tumor grade	3829/1976		
Grade 1		1.00 (ref)	
Grade 2		1.26 (1.04–1.53)	0.02
Grade 3		2.03 (1.68–2.44)	<0.001
Metastatic tumor subtype	4114/2145		
Luminal A-like		1.00 (ref)	
Luminal B-like		1.85 (1.65–2.08)	<0.001
HR + /HER2+		0.75 (0.63–0.90)	0.002
HR-/HER2+		0.96 (0.80–1.15)	0.65
HR-/HER2-		4.22 (3.74–4.75)	<0.001
No. of sites of disease	4189/2190	1.10 (1.07–1.12)	<0.001
Sites of met disease	4189/2190		
Bone		0.90 (0.82–0.98)	0.01
Brain		2.05 (1.78–2.36)	<0.001
Liver		1.49 (1.35–1.63)	<0.001
Lung		1.20 (1.08–1.33)	0.001
Other		1.10 (1.01–1.19)	0.03
Lines of treatment	4189/2190		
Any ET		0.61 (0.56–0.67)	<0.001
Any CDK		0.66 (0.61–0.72)	<0.001
Any chemotherapy		3.02 (2.23–4.01)	<0.001
Any radiation		1.08 (0.99–1.18)	0.07
Germline mutations^b^			
*BRCA1*	1456/671	1.61 (1.05–2.46)	0.03
*BRCA2*	1460/672	1.237 (0.82–1.85)	0.33
*CHEK2*	863/353	0.97 (0.56–1.65)	0.90

*CDK* cyclin-dependent kinase, *CI* confidence interval, *dx* diagnosis, *ET* endocrine therapy, *HER2* human epidermal growth factor receptor 2, *HR* *+* hormone receptor positive, *HR−* hormone receptor negative, *HR* hazard ratio, *MBC* metastatic breast cancer, *OS* overall survival, *PV* pathogenic variant, *y* years.

^a^ Total number and deaths differs for each univariable analysis based on variable missingness.

^b^ Germline mutations are assessed individually as presence of PV (yes/no) in *BRCA1*, *BRCA2*, or *CHEK2*. N overall includes only those tested for each mutation. HRs are calculated among individuals with known germline testing.

### Multivariable associations of age and clinical factors with OS

Age ≤35 years was associated with 1.2-times the hazard of death compared to age 45–55 years (*p* = 0.05) after adjustment for DFI, primary tumor grade, metastatic tumor subtype, and sites of disease (number and type) (Table 3). Older age (65–75 years) at diagnosis was also associated with shorter OS (HR [95% CI] v. 45–55 y = 1.15 [1.00–1.33], *p* = 0.05) (Table 3). All clinical factors significantly associated with OS in univariable associations remained significant in multivariable models. When using broader age categories and applying age ≤40 years as the cutoff for young diagnosis, multivariable models showed suggestive, but non-statistically significant associations between age ≤40 years at MBC and OS (HR [95% CI] v. 40–55 years = 1.13 [0.98–1.30], *p* = 0.08) (Table S2).

**Table 3:** Multivariable associations between age at MBC diagnosis and OS (*N* = 3754, Deaths = 1948)

Variable^a^	HR (95% CI)	*p*-value
Age group at MBC diagnosis^b^		
≤35 y	1.22 (1.00–1.48)	0.05
>35–45 y	0.94 (0.83–1.07)	0.36
>45–55 y	1.00 (ref)	
>55–65 y	0.93 (0.82–1.04)	0.20
>65- <75 y	1.15 (1.00–1.33)	0.05
Disease free interval		
de novo	1.00 (ref)	
>0–24 months	2.18 (1.89–2.52)	<0.001
≥24 months	1.22 (1.08–1.38)	0.001
Primary tumor grade		
Grade 1	1.00 (ref)	
Grade 2	1.21 (1.00–1.48)	0.05
Grade 3	1.45 (1.18–1.80)	0.001
Metastatic tumor subtype		
Luminal A-like	1.00 (ref)	
Luminal B-like	1.47 (1.27–1.69)	<0.001
HR + /HER2+	0.59 (0.48–0.73)	<0.001
HR−/HER2+	0.69 (0.55–0.85)	<0.001
HR−/HER2−	2.98 (2.54–3.50)	<0.001
No. of sites of disease	1.07 (1.04–1.10)	<0.001
Sites of disease		
Bone	1.21 (1.09–1.33)	<0.001
Brain	1.66 (1.43–1.94)	<0.001
Liver	1.71 (1.55–1.89)	<0.001
Lung	1.10 (0.98–1.23)	0.11

*CI* confidence interval, *HER2* human epidermal growth factor receptor 2, *HR* *+* hormone receptor positive, *HR−* hormone receptor negative, *HR* hazard ratio, *MBC* metastatic breast cancer, *OS* overall survival, *y* years.

^a^ All variables mutually adjusted.

^b^ (Deaths/N) by age group: ≤35 y (131/240), >35–45 y (372/713), >45–55 y (596/1116), >55–65 y (542/1077), >65–75 y (307/608).

### Association of age and OS by molecular subtype

In multivariable models stratified by metastatic molecular subtype, compared to older patients, OS was significantly shorter for younger patients (defined as both ≤35 years and ≤40 years at MBC diagnosis) with HER2-, luminal-B like tumors (N = 1213, deaths = 682) (Table 4). Age ≤35 years was associated with shorter OS for patients with hormone receptor-positive (HR + )/HER2+ tumors. Young age was not associated with OS in the other tumor subtypes.

**Table 4:** Multivariable associations between age at MBC diagnosis and OS within individual MBC tumor subtypes

Tumor subtype	N/Deaths	Age comparison	HR (95% CI)^a^	*p*-value
Luminal A	1030/436	≤35 y v. >35 y	1.33 (0.76–2.32)	0.32
		≤40 v. >40 y	0.83 (0.58–1.20)	0.33
Luminal B	1213/682	≤35 y v. >35 y	1.38 (1.03–1.86)	0.03
		≤40 v. >40 y	1.29 (1.04–1.60)	0.02
HR + /HER2+	386/143	≤35 y v. >35 y	2.00 (1.18–3.41)	0.01
		≤40 v. >40 y	1.06 (0.69–1.63)	0.78
HR–/HER2+	299/132	≤35 y v. >35 y	0.91 (0.52–1.61)	0.76
		≤40 v. >40 y	0.86 (0.56–1.32)	0.59
HR–/HER2−	826/555	≤35 y v. >35 y	1.08 (0.77–1.54)	0.65
		≤40 v. >40 y	1.08 (0.85–1.37)	0.51

*CI* confidence interval, *HER2* human epidermal growth factor receptor 2, *HR* *+*  hormone receptor positive, *HR−* hormone receptor negative, *HR* hazard ratio, *MBC* metastatic breast cancer, *OS* overall survival, *y* years.

^a^ All models adjusted for primary tumor grade (0/1, 2, 3), time between primary and metastatic diagnosis (de novo, >0–2 years, >=2 years), number of metastatic disease sites, and presence of metastases in bone lung, liver and brain (y/n for each). Bolded values are statistically significant at *p* < 0.05.

### Excluding individuals without direct metastatic tumor subtyping

In a sensitivity analysis excluding individuals without direct metastatic tumor subtyping and adjusting for receptor subtype only (HR + /HER2−, HR + /HER2 + , HR−/HER2−, HR−/HER2−), younger and older age were both associated with shorter OS (HR [95% CI] ≤35 years v. 45–55 years: 1.30 [1.03–1.65], *p* = 0.03; HR [95% CI]) >65- < 75 years v. 45−55 years: 1.21 [1.03−1.41], *p* = 0.02) (Table S3).

### Inclusion of individuals ≥ 75 years at MBC diagnosis

The association between young age and OS remained similar and statistically significant when including all individuals in the Ending Metastatic Breast Cancer for Everyone (EMBRACE) program regardless of age (range age 18–95 years) at MBC diagnosis (HR [95% CI] <=35 years v. 45–55 years: 1.21 [1.00–1.47], *p* = 0.05) (Table S4). As expected, the negative impact of older age (>65 years v. 45–55 years) on OS was strengthened when including patients 75 years and older. Other covariates in the multivariable Cox model had similar effect estimates compared with the main analysis.

### One-year OS

Out of 2190 deaths, 405 occurred within the first year after MBC diagnosis (≤40 years at MBC diagnosis: *N* = 305, 40–55 years: *N* = 914, >55– < 75 years: *N* = 971). In univariable models, younger age was associated with shorter one-year survival (HR [95% CI] ≤40 years v. >40–55 years=1.34 [1.02–1.76], *p* = 0.04) (Table S5), though this association disappeared after adjusting for clinical factors (HR [95% CI] ≤40 years v. >40–55 years=1.16 [0.87–1.53], *p* = 0.32) (Table S6). We were unable to assess the contribution of age ≤35 years on one-year survival in adjusted models given the limited number of events (*N* = 37 deaths in one year). Metastatic tumor subtype and DFI had an even greater impact on one-year survival compared to overall survival (Table S6 v. Table S2). Compared to patients with de novo disease, having a recurrent MBC diagnosed within 2 years of primary tumor diagnosis was associated with 3.7-times hazard of death (HR [95% CI] = 3.71 [2.64–5.22], *p* < 0.001). After adjusting for other clinical factors, compared to luminal A-like subtypes, both HER2−, luminal B-like MBC and HR−/HER2− disease were associated with higher hazard of death in one-year (HR [95% CI] luminal B-like= 2.06 [1.26–3.36], *p* = 0.004; HR−/HER2− = 6.27 [3.85–10.2], *p* < 0.001) (Table S6).

### Changes in tumor molecular subtype and OS

Among patients with recurrent MBC, those who experienced a change in tumor subtype from primary to first metastatic site (*N* = 392, 18%) were less likely to have primary lobular histology (7% vs. 13%, *p* < 0.001), more likely to have a higher primary grade (grade 3: 56% vs. 47%, *p* < 0.001), less likely to have received CDK4 inhibitors (26% vs. 56%, *p* < 0.001) and more likely to have received chemotherapy (99% vs. 95%, *p* < 0.001) in the metastatic setting compared to those who had a consistent subtype from primary to metastatic diagnosis. The most common subtype change in this cohort was from HR + /HER2− to hormone receptor-negative (HR−)/HER2− (7.3%) (Table S7). When testing subtype changes individually in a multivariable model adjusted for age at MBC diagnosis, DFI, primary tumor grade, and number and type of metastatic disease, the change from HR + /HER2− to HR−/HER2− disease was associated with shorter OS (HR [95% CI] vs. no change=1.71 [1.37-2.14], *p* < 0.001) (Table S8). The negative impact of hormone receptor change from positive to negative was not seen among individuals who maintained HER2+ status (HR [95% CI] for HR + /HER2+ to HR−/HER2+ vs. no change = 0.58 [0.38–0.88], *p* = 0.01). A change from HR + /HER2− to HR + /HER2+ subtype was also associated with improved OS (HR = 0.59 [0.35–0.99], *p* = 0.05).

## Discussion

In this study of over 4000 patients with de novo and recurrent MBC, age ≤35 years at MBC diagnosis was associated with worse OS after adjustment for clinicopathological prognostic features, compared to middle-age (45–55 years at diagnosis). The relationship between young age and OS appeared to be dependent on tumor molecular subtype, with significant associations observed among individuals with HER2−, luminal B-like metastatic tumors. Ultimately, this study illustrates the existence of a complex relationship between age and survival among patients with MBC that requires further investigation of the potential biologic and treatment/access to care mechanisms at play.

Studies have consistently shown that younger patients with de novo MBC fare better than their older counterparts^15,16,25^. This finding should not be conflated with patients with recurrent MBC, given that de novo MBC is more common among younger patients (accounting for 7.9% of incident BC cases among those aged 15–39 years compared to 5.5% among those 40–75 years)^26^, and evidence that among young women ( <40 years at primary diagnosis), those with de novo disease fare much better than those with recurrent MBC^27^. While several studies to date investigating age-survival relationships in patients with MBC have incorporated recurrent patients, either alone or alongside de novo patients, our ability to compare and draw conclusions from these studies is limited by key differences in patient populations, age-groups compared, and variations in adjustment factors of survival models.

First, the majority of studies include elderly patients ( >75 years), who often have a population-based average life-expectancy that is less than the follow-up time of the study, which may bias results. In addition, decisions on how to model age within survival models heavily influence study conclusions. A handful of studies incorporating patients with recurrent MBC have reported better OS among young age groups in multivariable analyses adjusting for demographic and clinicopathological factors^9,28–30^, seemingly conflicting with our findings; however, the use of dichotomized age ( < 50 v. ≥50 years,^28^ <65 v. ≥65 years^30^, <70 v. ≥70 years^9^), or continuous age^31^ likely masks true patterns of survival among younger patient groups.

Among studies that have assessed the association between finer age groups and survival following MBC diagnosis, most suggest a similar survival experience for younger patients compared to those in middle-age, conflicting with our results. However, these differences may be explained by differences in age group comparisons (with varied definitions of “young age”) and adjustment variables between studies. A large study among 14,403 women diagnosed with MBC at French cancer centers (ESME cohort) between 2008–2014 reported better OS for younger compared to older and middle-aged patients after adjusting for DFI, number and type of metastatic sites, and primary or metastatic tumor subtype^17^. However, this study compared age groups of <40 years v. 40-60 years v. >60 years. Given that we found ≤35 years as the group with increased risk for poor OS, assessing very young age may be of higher importance. Median survival between younger and middle-aged patients in the ESME study was very similar ( <40 years: 38.8 months v. 40-60 years = 38.4 months), matching our findings. Other studies reporting no association between young age and OS following MBC did not look at very young age ( ≤35 years)^10,32^, and/or lacked key adjustment factors^10,31^. For instance, though a SEER study of patients with recurrent MBC^31^ found no association between continuous age and OS, the model lacked adjustment for two key factors that are consistently strongly associated with survival: HER2 status and site of metastatic disease. These factors were also not incorporated in an analysis of 3447 patients enrolled in the ECOG trials who were diagnosed with BC between 1978–2002, where age <40 years at recurrent MBC diagnosis was not significantly associated with survival compared to other groups (40–49, 50–59, 60–69, >69 years)^10^. Overall, given the differences in existing studies and lack of assessment of very young individuals ( ≤35 years), it is plausible that our findings, though seemingly in conflict with the current literature, represent a true association between very young age and survival among patients with MBC.

Tumor molecular subtype consistently appears as a significant factor in projecting OS for patients with MBC in the clinical setting. As demonstrated within the EMBRACE cohort and supported by previous studies, the association between age and OS following metastatic diagnosis also varies by molecular subtypes. Age has less impact on OS among individuals with triple-negative breast cancer (TNBC), seen in our study and in prior literature. Two studies of patients with TNBC diagnosed after 2000, found no differences in OS for patients aged <75 years and ≥75 years^33^, and patients aged >50 years v. ≤50 years^29^. It is known that patients with de novo metastatic TNBC do worse than other subtypes^34^, and high distant recurrence rates within the first five years of primary diagnosis (estimated as high as 12% in one Dutch study) contribute to decreased survival in this patient group^4^. While previous studies have shown better survival for younger vs. older patients with HER2+ disease^29,35^, we found shorter OS among patients ≤35 years at diagnosis with HER2+ tumors. Longer OS among young patients with HER2+ tumors is expected due to their ability to take advantage of advances in HER2 therapy over the past 2 decades that extend survival compared to other subtypes^36^. However, our finding suggests that factors aside from treatment differences influence OS differences in MBC patients by tumor subtype.

Categorization of HR + /HER2− tumors into luminal A-like v. luminal B-like revealed further differences in OS by age. Compared to luminal A tumors, luminal B tumors are characterized by a higher expression of genes associated with tumor cell proliferation^37^, and are associated with worse prognosis overall^38^. In the context of early BC, luminal B tumors may indicate higher likelihood of recurrence, providing one theory for this observed worse prognosis^39^. Interestingly, in this study young age was consistently associated with poorer overall survival among patients with HER2− luminal B-like tumors even after adjustment for DFI (which captures de novo v. recurrent status). Thus, the difference in survival among HER2− luminal B-like patients cannot be fully explained by a higher number of recurrent luminal B v. luminal A tumors. In general, our finding of strong associations between metastatic tumor subtype and OS makes the case for incorporating such metastatic tumor data into prognostic models, especially given that our study was able to assess subtype in metastatic tumors samples for the majority (76%) of patients, while most studies have relied on primary tumor subtyping.

Our finding of consistently worse OS with greater number and particular sites of disease at diagnosis of MBC has been reported elsewhere^10,17^. Before and after adjustment for other clinical factors, the presence of brain or liver metastases portended considerably worse OS, also consistent with existing literature showing up to 4x increased risk for those with liver metastases and up to 15x increased risk for those with brain metastases^28^.

Subtype switching between primary and metastatic tumor estrogen receptor (ER), progesterone receptor (PR), and HER2 status has been reported elsewhere. One review article combining data from many studies estimated average rates of discordance to be 14% (ER), 21% (PR), and 10% (HER2)^40^, though there is wide variation in estimates between studies. Overall, subtype switches resulting in loss of positivity are most common^40–42^, which was confirmed in EMBRACE. Our finding that women who had a primary HR+ tumor that switched to an HR− MBC tumor had worse OS aligns with findings from other studies^41,43–46^; the reduction in survival may be attributed to historically fewer therapeutic options for patients with HR− tumors or an associated increase in the biologic aggressiveness of the HR- disease. Overall, conclusions based on subtype switches indicate a need to re-assess the phenotype of the metastatic tumor at diagnosis of MBC at a minimum in order to provide proper treatment for patients of all ages.

The strengths of this study include the large sample size and robust data collection with detailed information on patient tumor subtypes, treatment regimens, and outcomes. However, several study limitations must be considered when interpreting our findings. First, we did not include specific treatment regimens in our models given the vast diversity of regimens used in treating patients with MBC and the fact that many MBC treatments are decided based on patient response to primary treatments. Second, though we used metastatic tumor subtype as the main adjustment factor here, we were unable to obtain metastatic tumor subtype on 924 (24%) of patients and instead relied on primary subtype for these patients. However, when we restricted analysis to only those patients with direct metastatic tumor subtyping available, associations between age at diagnosis and OS remained similar. Because we did not have direct gene expression profiling available for MBC or primary tumors, nor did we have Ki67 status on the vast majority of patients, we used inferred molecular subtype based on clinicopathologic characteristics (tumor grade and PR positivity). While this captures some of the variation in HER2− luminal A v. luminal B tumor subtypes, and immunohistochemistry (IHC)-based criteria have been used across the literature in place of genetic profiling^39^, this remains an imperfect surrogate. In addition, inferred subtypes were by necessity defined in part by primary tumor grade, which may have resulted in misclassification of subtypes. Thus, further studies with direct molecular subtyping for metastatic tumors are needed to confirm our results.

In this large observational study, patients with MBC diagnosed at a very young age ( ≤35 years) experienced shorter OS compared to those diagnosed in middle-age, especially when comparing individuals with HER2−, luminal B-like metastatic tumors. The mechanisms behind this association are unclear. Our findings, which reflect observational data, should not impact current consensus guidelines that young age should not be a reason to treat MBC with more aggressive therapy^47–49^. Moreover, our study emphasizes that tumor subtype remains the most important predictor of OS alongside initial metastatic disease site(s). While promising advances have been made to decrease BC mortality in the U.S., with a 58% reduction in mortality reported between 1975 and 2019, the majority of this decline is due to treatment for early-stage BC or screening, as opposed to MBC treatment^50^, highlighting the importance of continuing to study this patient subgroup and find biologic as well as clinical explanations for survival advantages to identify avenues to improve survival.

## Methods

### Cohort

We used data from a prospectively maintained institutional database (EMBRACE), that includes women treated for MBC at Dana-Farber Cancer Institute who consented and enrolled to one of the following Dana-Farber/Harvard Cancer Center protocols: 09-204,05-246, 93-085, 11-104 and/or 17-000. Data collected includes patient demographics, clinicopathological characteristics, biopsy type, details on surgery and metastatic treatment regimens, sites of disease, and patient outcomes. Survival follow-up is collected via medical record review and via linkage to the National Death Index. For this analysis, eligible patients included those enrolled from January 2009 to December 2023 (N = 4397), who were <75 years at MBC diagnosis, with follow-up through 2024. Patients ≥75 years were excluded from the primary analysis due to the shorter projected lifespan of this group from comorbidities and competing causes of death. All protocols were approved by the Dana-Farber/Harvard Cancer Center institutional review board.

### Tumor molecular subtype

Primary and metastatic tumor molecular subtype were determined by review of pathology reports, and classified based on ER, PR, and HER2 staining. Tumors were considered HR+ if ≥1% staining for ER or PR. HER2 status was assigned according to ASCO/CAP guidelines (HER2+ defined as 3+ by IHC, HER2 copy number ≥6, or HER2/CEP17 ratio ≥ 2.0)^51^. Where metastatic tumor molecular subtype was not available (i.e., the initial site of metastatic disease was not biopsied or biopsy was too small to assess subtype), subtype was assumed to be the same as that of the primary tumor. HR + /HER2− metastatic tumors were further characterized by inferring luminal A-like or luminal B-like subtype. Tumors were classified as HER2− luminal B-like if the tumor was grade 3 and/or PR staining was <10% (considered “PR low”). Due to difficulties in grading metastatic tumors, primary tumor grade was used for this classification. PR was directly measured in metastatic tumors in most cases; where this was not possible, PR staining in the primary tumor was used to designate luminal A and B-like subtypes.

### Statistical analysis

Characteristics of cohort participants were described with medians and IQR or frequency (%). OS was examined from the time of MBC diagnosis, using Kaplan-Meier curves, stratified by age group at MBC diagnosis ([1] ≤40 years, 41–55 years, >55- < 75 years, [2] ≤35, 36–40, 41–45, 46–55, >55–65, >65- < 75 years), and inferred metastatic tumor molecular subtype. Two definitions were chosen for age group at diagnosis to enable direct comparison of our results with prior studies (most often using ≤40 years as the young cutoff), while also providing novel information on the youngest group of patients. Univariable Cox regression models estimated HRs and 95% CIs for OS by clinical features: including germline mutation status, primary tumor stage and grade, metastatic tumor subtype, DFI, number and type of metastatic sites developed during follow-up (yes/no for bone, brain, liver, lung, other), number of metastatic treatment regimens, and receipt of endocrine therapy (ET), cyclin-dependent kinase 4/6 (CDK4/6) inhibitor therapy, chemotherapy and/or radiation at any time during metastatic treatment. The association between age group at MBC diagnosis and OS was tested in a multivariable Cox model, after adjusting for tumor factors: metastatic tumor molecular subtype (inferred), primary tumor grade (0/1, 2, 3), DFI (defined as: de novo, >0–2 y, ≥2 years), and number and type of metastatic sites at the time of MBC diagnosis. Because inferred metastatic tumor molecular subtype was defined in part using primary tumor grade, we tested the correlation between these variables (all <0.15, considered acceptably low to avoid collinearity). Multivariable models assessing the association between age and OS were also stratified by metastatic tumor subtype. In addition to OS, we tested all models using one-year survival as the outcome of interest.

We conducted several sensitivity analyses. First, to assess the influence of excluding older MBC patients, we repeated analyses without restricting to <75 years at diagnosis. Second, we repeated analyses excluding individuals without tumor subtyping directly available from a metastatic sample. We then assessed differences in clinical and patient characteristics between individuals with recurrent MBC who experienced a change in molecular subtype from primary to metastatic tumor compared to those who did not experience a change, and calculated frequencies of specific tumor subtype changes. The association between specific subtype changes and OS were tested in multivariable Cox models, adjusting for age at MBC diagnosis.

## Supplementary information

Supplementary Information

## Data Availability

The data that support the findings of this study are not openly available due to reasons of sensitivity (e.g., inclusion of indirect patient identifiers such as age, sex, ethnicity, and treatment location) but are available from the corresponding author upon reasonable request and completion of a data use agreement. Data are located in controlled access data storage as part of the IRB-approved, Breast Cancer Data Warehouse at Dana-Farber Cancer Institute. Code availability statement: All coding was done using standard procedures in STATA version 18.0. Coding is specific to the dataset and is available from the corresponding author upon reasonable request.

## Code availability

All coding was done using standard procedures in STATA version 18.0. Coding is specific to the dataset and is available from the corresponding author upon reasonable request.
