# Supplementary material for: The association between young age at metastatic breast cancer diagnosis and overall survival in the EMBRACE study
Source: NPJ Breast Cancer. 2025 Aug 29;11:96. doi: 10.1038/s41523-025-00822-y (PMC12397373; doi:10.1038/s41523-025-00822-y)
Supplement: Supplementary file 1 — Supplementary Information [file 41523_2025_822_MOESM1_ESM.pdf]

SUPPLEMENT

The association between young age at metastatic breast cancer diagnosis and overall survival in the EMBRACE study

Tables: 8

Table S1. Patient and Tumor Characteristics of women ≤40y at diagnosis in EMBRACE cohort (N=571)

Variable[1]	Total (N=571)	≤35 y (N=260)	>35-40y (N=311)
Died	304 (53%)	143 (55%)	162 (52%)
Time from met dx to death or censorship, median (IQR)	2.3 (0.9-4.6)	2.2 (0.9-3.6)	2.3 (1.1-4.0)
Germline PV testing done	370 (65%)	171 (66%)	199 (64%)
Known PVs[2]			
ATM	2 (0.5%)	1 (<1%)	1 (<1%)
BRCA1	9 (2%)	4 (2%)	5 (2%)
BRCA2	9 (2%)	8 (3%)	1 (<1%)
CHEK2	3 (0.8%)	2 (1%)	1 (<1%)
PALB2	2 (0.5%)	1 (<1%)	1 (<1%)
TP53	3 (0.8%)	3 (1%)	0 (0%)
Other PV	9 (2%)		
Age at dx, median (IQR)	34 (30-36)	30 (28-32)	36 (35-38)
Stage			
0	4 (1%)	3 (1%)	1 (<1%)
1	35 (6%)	10 (4%)	25 (8%)
2	170 (30%)	76 (29%)	94 (30%)
3	150 (26%)	66 (25%)	84 (27%)
4 (*de novo*)	204 (36%)	101 (39%)	103 (33%)
Missing stage	8 (1%)	4 (2%)	4 (1%)
Histology			
DCIS	6 (1%)	4 (2%)	2 (1%)
Invasive ductal	476 (83%)	217 (83%)	259 (83%)
Invasive lobular	29 (5%)	9 (3%)	20 (6%)
Tubular	0 (0%)	0 (0%)	0 (0%)
Mucinous	2 (0%)	1 (<1%)	1 (<1%)
Micropapillary	1 (0%)	1 (0%)	0 (0%)
Mixed (IDC and ILC)	28 (5%)	12 (5%)	13 (4%)
Missing/Other	29 (5%)	16 (6%)	13 (4%)
Tumor grade			
1	12 (2%)	1 (<1%)	11 (4%)
2	161 (28%)	65 (25%)	96 (31%)
3	367 (64%)	177 (68%)	190 (61%)
Missing	31 (5%)	17 (7%)	14 (5%)
Subtype			
Luminal A-like[3]	103 (18%)	33 (13%)	70 (23%)
Luminal B-like[4]	181 (32%)	87 (33%)	94 (30%)
HR+/HER2+	102 (18%)	53 (20%)	49 (16%)
HR-/HER2+	63 (11%)	32 (12%)	31 (10%)
HR-/HER2-	109 (19%)	48 (18%)	61 (20%)
Missing	13 (2%)	7 (3%)	6 (2%)
**Metastatic tumor and treatment characteristics**			
Age at met dx, median (IQR)	36 (32-38)	32 (30-34)	38 (37-39)
Year of met dx, median (range)	2016 (1996-2023)	2016 (1996-2023)	2015 (2002-2022)
Years from primary to metastatic dx, median (IQR)	1.2 (0-2.7)	0.9 (0-2.6)	1.4 (0-2.8)
Subtype[5]			
Luminal A-like[3]	104 (18%)	34 (13%)	70 (23%)
Luminal B-like[4]	172 (30%)	87 (33%)	85 (27%)
HR+/HER2+	91 (16%)	45 (17%)	46 (15%)
HR-/HER2-	70 (12%)	35 (13%)	35 (11%)

HR-/HER2-	128 (22%)	55 (21%)	73 (23%)
Missing	6 (1%)	4 (2%)	2 (1%)
Subtype different from primary subtype[6]	45 (19%)	20 (21%)	25 (18%)
Lines of therapy from dx to last follow-up, median (IQR)	3 (1-6)	3 (1-6)	3 (1-6)
Types of metastatic therapy received, from dx to last follow-up[7], median (IQR)			
ET	339 (59%)	160 (51%)	160 (51%)
CDK 4/6i	230 (40%)	160 (51%)	160 (51%)
Chemotherapy	541 (95%)	160 (51%)	160 (51%)
Radiation	293 (51%)	160 (51%)	160 (51%)
Number of metastatic sites, from dx to last follow-up, median (IQR)	4 (3-5)	4 (3-5)	4 (3-5)
Sites of metastatic disease anytime from dx to last follow-up[8], median (IQR)			
Bone	314 (55%)	140 (54%)	174 (56%)
Brain	72 (13%)	30 (12%)	42 (14%)
Liver	188 (33%)	86 (33%)	102 (33%)
Lung	108 (19%)	54 (21%)	54 (17%)
Other[9]	310 (54%)	141 (54%)	169 (54%)

1 Variable described by N (%) if not specified

2 Includes pathogenic or likely pathogenic variants. Percent out of N with any germline testing in age group

3 HR+/HER2- with primary tumor grade <3 AND met tumor PR staining >=10%

4 HR+/HER2- with primary tumor grade=3 OR met tumor PR staining <10%

5 When metastatic tumor subtyping unavailable, primary tumor subtype used (N=390, ≤35 y: 168, ≤40 y: 222)

6 Among those with recurrent MBC and both primary and metastatic tumor subtyping available (N=238)

7 Multiple types of therapy are possible for one patient

8 Multiple sites of disease are possible for one patient

9 Other sites reported include: ovary (1), skin of breast (1)

CDK4/6i= cyclin-dependent kinases 4 and 6 inhibitor, DCIS=ductal carcinoma in situ, dx=diagnosis, ET=endocrine therapy, HER2=human epidermal growth factor receptor 2, HR+=hormone receptor positive, HR-=hormone receptor negative, IDC=invasive ductal carcinoma, ILC=invasive lobular carcinoma, IQR=interquartile range, PR=progesterone receptor, PV=pathogenic variant, y=years

Table S2. Multivariable associations between age at MBC diagnosis and OS, 40 y as young cut off (N=3,754, Deaths=1,948)

Variable[1]	HR (95% CI)	p-value
Age group at MBC diagnosis[2]		
≤40 y	1.13 (0.98-1.30)	0.08
>40-55 y	1.00 (ref)	
>55-<75y	1.03 (0.93-1.13)	0.60
Disease-free interval		
de novo	1.00 (ref)	
>0-24 months	2.19 (1.90-2.53)	<0.001
≥24 months	1.23 (1.09-1.38)	0.001
Primary tumor grade		
Grade 1	1.00 (ref)	
Grade 2	1.20 (0.99-1.46)	0.07
Grade 3	1.43 (1.16-1.77)	0.001
Metastatic tumor subtype		
Luminal A-like	1.00 (ref)	
Luminal B-like	1.47 (1.27-1.70)	<0.001
HR+/HER2+	0.59 (0.48-0.72)	<0.001
HR-/HER2+	0.68 (0.55-0.84)	<0.001
HR-/HER2-	2.96 (2.52-3.48)	<0.001
No. of sites of disease	1.07 (1.04-1.09)	<0.001
Sites of disease		
Bone	1.21 (1.10-1.33)	<0.001
Brain	1.62 (1.39-1.89)	<0.001
Liver	1.70 (1.54-1.88)	<0.001
Lung	1.11 (0.99-1.24)	0.08

1 All variables mutually adjusted

2 (Deaths/N) by age group: ≤40y (286/535), >35-55y (813/1534), >55-75y (849/1685)

CI=confidence interval, HER2=human epidermal growth factor receptor 2, HR+=hormone receptor positive, HR-=hormone receptor negative, HR=hazard ratio, MBC=metastatic breast cancer, OS=overall survival, y=years

Table S3. Multivariable associations between age at MBC diagnosis and OS based on direct subtyping of metastatic tumor (N=2,864, Deaths=1,484)

Variable[1]	HR (95% CI)	p-value
Age group at MBC diagnosis		
≤35 y	1.30 (1.03-1.64)	0.03
>35-45y	0.92 (0.79-1.07)	0.27
>45-55y	1.00 (ref)	
>55-65y	0.93 (0.81-1.06)	0.27
≥65y-<75y	1.21 (1.03-1.41)	0.02
Disease-free interval		
de novo	1.00 (ref)	
>0-24 months	2.56 (2.16-3.04)	<0.001
≥24 months	1.28 (1.12-1.48)	<0.001
Primary tumor grade		
Grade 1	1.00 (ref)	
Grade 2	1.24 (0.99-1.55)	0.07
Grade 3	1.77 (1.41-2.23)	<0.001
Metastatic tumor subtype		
HR+/HER2-	1.00 (ref)	
HR+/HER2+	0.47 (0.38-0.58)	<0.001
HR-/HER2+	0.57 (0.46-0.70)	<0.001
HR-/HER2-	2.26 (1.98-2.59)	<0.001
No. of sites of disease	1.07 (1.04-1.10)	<0.001
Sites of disease		
Bone	1.23 (1.04-1.10)	<0.001
Brain	1.63 (1.36-1.96)	<0.001
Liver	1.84 (1.64-2.06)	<0.001
Lung	1.04 (0.92-1.19)	0.50

1 All variables mutually adjusted in multivariable model

2 (N/Deaths) by age group: ≤35y (86/157), >35-45y (284/544), >45-55y (447/842), >55-65y (428/859), >65-75y (239/462)

CI=confidence interval, HER2=human epidermal growth factor receptor 2, HR+=hormone receptor positive, HR-=hormone receptor negative, HR=hazard ratio, MBC=metastatic breast cancer, OS=overall survival, y=years

Table S4. Multivariable associations between age at MBC diagnosis and OS, all ages in EMBRACE (N=3,908, Deaths=2,019)

Variable[1]	HR (95% CI)	p-value
Age group at MBC diagnosis[2]		
≤35 y	1.21 (1.00-1.47)	0.05
>35-45 y	0.94 (0.82-1.07)	0.33
>45-55 y	1.00 (ref)	
>55-65y	0.93 (0.82-1.04)	0.21
>65 y	1.20 (1.05-1.37)	0.01
Disease-free interval		
de novo	1.00 (ref)	
>0-24 months	2.15 (1.86-2.47)	<0.001
≥24 months	1.19 (1.06-1.34)	0.004
Primary tumor grade		
Grade 1	1.00 (ref)	
Grade 2	1.24 (1.02-1.50)	0.03
Grade 3	1.47 (1.20-1.81)	<0.001
Metastatic tumor subtype		
Luminal A-like	1.00 (ref)	
Luminal B-like	1.45 (1.26-1.67)	<0.001
HR+/HER2+	0.58 (0.47-0.71)	<0.001
HR-/HER2+	0.68 (0.55-0.84)	<0.001
HR-/HER2-	2.92 (2.50-3.42)	<0.001
No. of sites of disease	1.07 (1.04-1.09)	<0.001
Sites of disease		
Bone	1.18 (1.08-1.30)	<0.001
Brain	1.65 (1.42-1.93)	<0.001
Liver	1.71 (1.55-1.89)	<0.001
Lung	1.10 (0.98-1.23)	0.10

1 All variables mutually adjusted

2 (Deaths/N) by age group: ≤35y (131/240), >35-45 y (372/713), >45-55 y (596/1,116), >55-65 y (542/1,077), >65-75 y (378/762)

CI=confidence interval, HER2=human epidermal growth factor receptor 2, HR+=hormone receptor positive, HR-=hormone receptor negative, HR=hazard ratio, MBC=metastatic breast cancer, OS=overall survival, y=years

Table S5. Univariable associations between clinical variables and one-year survival (N=4,189, Deaths=405)

Variable	N / Deaths[1]	HR (95% CI)	p-value
Age group at MBC diagnosis	4,189 / 405		
≤40 y		1.34 (1.02-1.76)	0.04
>40-55 y		1.00 (ref)	
>55y		0.85 (0.68-1.05)	0.13
Disease-free interval	4,189 / 405		
de novo		1.00 (ref)	
>0-24 months		7.28 (5.21-10.3)	<0.001
≥24 months		1.66 (1.18-2.32)	0.003
Primary tumor subtype	3,982/ 419		
Luminal A-like		1.00 (ref)	
Luminal B-like		3.69 (2.54-5.35)	<0.001
HR+/HER2+		1.26 (0.71-2.23)	0.44
HR-/HER2+		2.27 (1.29-4.00)	0.004
HR-/HER2-		12.24 (8.59-17.5)	<0.001
Primary tumor stage	4,110/401		
In situ		1.00 (ref)	
Stage I		1.30 (0.40-4.19)	0.67
Stage II		2.12 (0.68-6.64)	0.20
Stage III		3.17 (1.01-9.94)	0.05
Stage IV (de novo)		0.78 (0.24-2.53)	0.68
Primary tumor grade	3,800/384		
Grade 1		1.00 (ref)	
Grade 2		1.65 (0.80-3.43)	0.18
Grade 3		5.76 (2.85-11.6)	<0.001
Metastatic tumor subtype	4,114/401		
Luminal A-like		1.00 (ref)	
Luminal B-like		3.43 (2.25-5.21)	<0.001
HR+/HER2+		0.80 (0.37-1.76)	0.58
HR-/HER2+		2.23 (1.22-4.07)	0.01
HR-/HER2-		14.3 (9.7-21.1)	<0.001
No. of sites of disease	4,189/405	1.10 (1.05-1.17)	<0.001
Sites of disease	4,189/405		
Bone		0.64 (0.52-0.78)	<0.001
Brain		3.67 (2.91-4.61)	<0.001
Liver		1.98 (1.62-2.42)	<0.001
Lung		1.99 (1.62-2.46)	<0.001
Other		1.24 (1.02-1.52)	0.03
Lines of treatment	4,189/405		
Any ET		0.33 (0.27-0.40)	<0.001
Any CDK		0.36 (0.28-0.45)	<0.001
Any chemotherapy		11.2 (3.58-34.7)	<0.001
Any radiation		0.82 (0.67-1.00)	0.05
Germline mutations[2]			
BRCA1	1,456/121	1.22 (0.45-3.30)	0.70
BRCA2	1,460/122	0.99 (0.41-2.44)	0.99
CHEK2	863/68	0.39 (0.05-2.78)	0.35

1 Total number and deaths differs for each univariable analysis based on variable missingness.

2 Presence of pathogenic variant (yes/no) in *BRCA1*, *BRCA2*, or *CHEK2*. HRs calculated among individuals with definitive germline testing for each gene.

CDK, cyclin-dependent kinase, CI=confidence interval, ET=endocrine therapy, HER2=human epidermal growth factor receptor 2, HR+=hormone receptor positive, HR-=hormone receptor negative, HR=hazard ratio, MBC=metastatic breast cancer, y=years

Table S6. Multivariable associations between age at MBC diagnosis and 1-year survival (N=3,754 Deaths=382)

Variable[1]	HR (95% CI)	p-value
Age group at MBC diagnosis		
≤40 y	1.16 (0.87-1.53)	0.32
>40-55y	1.00 (ref)	
>55-<75y	1.05 (0.84-1.32)	0.67
Disease-free interval		
de novo	1.00 (ref)	
>0-24 months	3.71 (2.64-5.22)	<0.001
≥24 months	1.37 (0.97-1.93)	0.07
Primary tumor grade		
Grade 1	1.00 (ref)	
Grade 2	4.0 (2.78-5.79)	<0.001
Grade 3	1.55 (1.07-2.24)	0.02
Metastatic tumor subtype		
Luminal A-like	1.00 (ref)	
Luminal B-like	2.06 (1.26-3.36)	0.004
HR+/HER2+	0.49 (0.21-1.16)	0.10
HR-/HER2+	1.10 (0.56-2.18)	0.78
HR-/HER2-	6.27 (3.85-10.2)	<0.001
No. of sites of disease	0.99 (0.92-1.05)	0.66
Sites of disease		
Bone	1.22 (0.98-1.05)	0.08
Brain	1.48 (1.18-1.86)	<0.001
Liver	2.36 (1.91-2.92)	<0.001
Lung	2.20 (1.71-2.82)	<0.001

1 All variables mutually adjusted in multivariable model

2 (Deaths/N) by age group: ≤40y (571/74), >40-55y (168/1,693), >55-75y (163/1,925)

CI=confidence interval, HER2=human epidermal growth factor receptor 2, HR+=hormone receptor positive, HR-=hormone receptor negative, HR=hazard ratio, MBC=metastatic breast cancer, y=years

Table S7. Frequency of molecular subtype changes from primary to metastatic diagnosis

Primary tumor subtype	Metastatic tumor subtype, N (%)[2]			
	HR+/HER2-	HR+/HER2+	HR-/HER2+	HR-/HER2-
HR+/HER2-		38 (1.8 %)	11 (0.5%)	157 (7.3%)
HR+/HER2+	50 (2.38%)		51 (2.4%)	11 (0.5%)
HR-/HER2+	4 (0.2%)	6 (0.3%)		18 (0.8%)
HR-/HER2-	36 (1.7%)	2 (0.1%)	8 (0.4%)	

1 Percent is calculated among all recurrent MBC patients with direct metastatic subtyping available (N=2,153)

HER2=human epidermal growth factor receptor 2, HR+=hormone receptor positive, HR-=hormone receptor negative, MBC=metastatic breast cancer

Table S8. Hazard Ratio for OS for tumor molecular subtype change from primary to metastatic dx, compared to no subtype change (N=2,881)[1]

Primary tumor subtype	Metastatic tumor subtype			
	HR+/HER2-	HR+/HER2+	HR-/HER2+	HR-/HER2-
	HR (95% CI)[2]			
HR+/HER2-		**0.59 (0.35-0.99)**	0.69 (0.17-2.80)	**1.71 (1.37-2.14)**
HR+/HER2+	1.18 (0.81-1.73)		**0.58 (0.38-0.88)**	0.79 (0.33-1.92)
HR-/HER2+	1.08 (0.27-4.36)	0.16 (0.02-1.13)		1.29 (0.73-2.28)
HR-/HER2-	1.37 (0.92-2.04)	0.97 (0.14-7.00)	0.91 (0.38-2.21)	

1 For those with both primary tumor and metastatic tumor subtyping available, N=2,881. Comparing each group to those with no subtype change (N=2,434)
2 HR examine specific subtype change v. no subtype change. Multivariable models adjusted for age at MBC diagnosis (≤40y, 40-55y, >55-75y), DFI, primary tumor grade, number and sites of metastatic disease
Bolded values signify statistical significance at p value <0.05
DFI=disease-free interval, HER2=human epidermal growth factor receptor 2, , HR+=hormone receptor positive, HR-=hormone receptor negative, HR=hazard ratio, MBC=metastatic breast cancer
